# Annotations

**Published:** 1909-03-06

**Authors:** 


### Annotations.

### The Organisation of Medical Practice.

Mr. Bernard Shaw, never inclined to hide his ideas under a bushel, gave on February 16 to the members of the Medico-Legal Society some Socialist criticisms of the economics of medical practice, which we summarised in this space a fortnight ago. The main objection we would bring against his portrayal of the evils of the present system is that it is rather overdrawn. Slum dispensaries, which we suppose are indicated when Mr. Shaw speaks of patients demanding and being supplied with "cheap cures," comprise quite a small proportion of existing practices. In reality there are few medical men at all resembling the astute alien in "The Doctor's Dilemma," who sold bottles of medicine for sixpence, "cure guaranteed." Certainly the general practitioner finds the sick public a hard master—and this is why so much competition exists for posts with a fixed salary; but the idea that the mere indefinite increase of such posts is of itself an absolute remedy for the faults of both sides is not one to commend itself to the experienced. Of this there is a hint in Mr. Shaw's admission that there would be private practitioners even after the doctor had been placed in the position of public officer of health. Indeed, the foolish old lady, to take an instance, who likes to imbibe drug decoctions all day long, might encounter a few more refusals to gratify her than she does at present; but it is unfortunately probable that in the end she would find someone to treat her on her own lines. And the democratic control spoken of might easily, considering the layman's natural but exorbitant demand for sympathy and humouring, prove not much less detrimental than the present pressure of patients' opinion. Mr. Shaw should have mentioned the importance of scientific education of the public.

### The Hunterian Oration.

In a year distinguished by the addition of his Royal Highness the Prince of Wales to the list of Honorary Fellows of the Royal College of Surgeons, that body has also had a Hunterian Oration a little out of the common. The difficulty of handling so worn a theme as the virtues of the commemorated surgeon must be very considerable, and Mr. Henry Morris is to be congratulated on the success with which he surmounted it. He took for text the position of John Hunter as a philosopher, which has already received the attention of the historian Buckle. With the conclusions of the latter Mr. Morris is, however, not prepared altogether to agree. Especially is this so with regard to the proportions and causes of the inductive and deductive elements of John Hunter's philosophical work. According to Buckle the latter style represents the inherited Scottish character, reinforced by the not very extensive education which the great surgeon received during boyhood in his native country; whereas the inductive method was grafted on to this by residence in London and contact during his professional career there with this especially English plan of seeking after truth. The President of the College disputes this view of Hunter's personality, and ascribes his success with pure inductive reasoning not to English influences, but to the natural bent of a great mind. This view is one which commends itself much more to our judgment than the somewhat forced explanation of the historian; and the oration in which it is so felicitously expounded may be commended to everyone interested in Baconian philosophy, whether he be of the medical profession or not. But there is a premiss in one of the syllogisms, granted both by Buckle and Mr. Morris, which may perhaps ultimately want qualification. This is the proposition that deduction is characteristic of the Scottish bent, induction of the English. We are, of course, well aware that induction was first advocated and expounded in England by Francis Bacon; but we demur to the idea that it is little practised north of the Tweed. After all the Lowlands of Scotland, whence most of the Scottish Philosophers have come, are populated by people indistinguishable ethnologically from the English and of essentially similar character. The Scots may lean more towards deductive reasoning than the English do, but it cannot be maintained that the more excellent way is a monopoly of South Britain.

### X-Ray Victims.

The distressing condition, physically and financially, to which a number of members of the medical profession and of radiographers not medically qualified have been reduced by the results of their work and their devotion to it has been for some time made known to the public in respect of one or two of the worst cases; and here and there the public conscience, touched by the story of these well-named "martyrs of science," has been generously responsive for their relief. There is always a danger that in cases of this sort the gratitude of the public may be unequal in its incidence; that the dramatic or sensational element which enters so largely into the composition of the modern newspaper article will lead to the benefit of some much more adequately than of others. It is therefore a great satisfaction to note that the *Standard* and the *Daily Express*, which are appealing for practical recognition of the sacrifices which their labours on behalf of humanity have entailed upon many radiographers, have compiled as complete a list as possible of the worst cases. Eight medical men and six other workers are included in this list, and details of their condition is given; fortunately not all have suffered as have Dr. Hall Edwards and one or two others, but all the cases are serious enough. It remains to be pointed out that these gentlemen's sufferings are due to the fact that they were among the earliest workers in the field, and that, warned by the terrible fate that has overtaken them, their successors have been able to adopt preventive measures of which originally the necessity was undreamt. The number of sufferers is therefore unlikely to increase; and this is all the more reason why the public, in whose interests they have suffered, should, as far as material comforts can go, compensate them liberally for their mutilations.

